# Rare micropupil secondary to congenital cataract surgery favoring the development of the affected eye: a case report

**DOI:** 10.1186/s12886-024-03507-5

**Published:** 2024-06-14

**Authors:** Zicheng Ma, Siquan Zhu

**Affiliations:** Department of Ophthalmology, Beijing AnZhen Hospital, Capital Medical University, Beijing, 100029 China

**Keywords:** Micropupil, Congenital cataract surgery, Complication, Pinhole imaging

## Abstract

**Background:**

Congenital microcoria has been extensively reported and usually leads to visual dysfunction or blindness. However, micropupil development secondary to cataract surgery has never been reported. Here, we describe a rare case of micropupil development in infancy that occurred secondary to combined cataract extraction and intraocular lens implantation for treatment of congenital cataract. When the patient reached adulthood, the affected eye not only gained good vision but also showed better ocular development and refractive status than the fellow eye.

**Case presentation:**

A 17-year-old boy presented to our outpatient clinic with decreased vision in his left eye related to congenital cataract surgery at 6 months of age. The affected eye had exhibited a pinhole pupil since the third month postoperatively. The condition had been managed with observation and regular monocular occlusion treatment. Upon presentation to our clinic, the best-corrected visual acuity (BCVA) in his fellow eye was 0.0 logMAR(20/20) with a refraction of − 5.75 diopters cylinder/−2.25 diopters sphere, and the BCVA in his affected eye was 0.5 logMAR(20/40) with a refraction of 0.00 diopters. Ophthalmic examination of the affected eye revealed a pinhole pupil (approximately 0.5 mm) with high light reflex sensitivity but no response to pupil-dilating drugs. The patient underwent pupilloplasty of the affected eye under corneal surface anesthesia. Postoperative examination revealed better ocular development in the affected eye than in the fellow eye (axial length: 24.21 vs. 27.02 mm, respectively) as well as better refractive status in the affected eye (BCVA of 0.0 logMAR(20/20) with a refraction of − 2.23 diopters cylinder/−3.00 diopters sphere vs. 0logMAR(20/20) with a refraction of -5.75 diopters cylinder/-2.25 diopters sphere).

**Conclusions:**

We have reported a rare case of micropupil development secondary to congenital cataract surgery, which is an uncommon complication, especially in children. However, unlike congenital microcoria, the secondary pinhole pupil may have reduced imaging haze and halos, possibly favoring the development of the affected eye. This case provides further insight into the treatment of congenital cataract.

**Supplementary Information:**

The online version contains supplementary material available at 10.1186/s12886-024-03507-5.

## Background

Many complications can occur after congenital cataract surgery, including posterior capsule opacification (PCO), aphakic or pseudophakic glaucoma, uveitis, pupillary displacement, and intraocular lens (IOL) decentration [1]. However, reports of postoperative pinhole pupil without clinically significant posterior iris adhesions are uncommon.

Consistent and appropriate development of ocular structures during childhood is important for good vision [2]. The pupil opens and closes in response to light intensity, thereby influencing the amount of light that reaches the retina. Congenital microcoria is an extremely rare autosomal dominant disease that affects iris development and hinders pupil function. It manifests as a pinhole pupil (< 2 mm) that causes visual disorders (e.g., hemeralopia and light hypersensitivity) through iris hypoplasia [3].

Here, we describe a patient who underwent congenital cataract surgery in one eye during infancy and developed a pinhole pupil (approximately 0.5 mm) secondary to the surgery; this condition was not treated. However, following regular monocular occlusion treatment, the patient exhibited relatively good ocular development in adulthood and maintained good visual acuity after pupilloplasty.

## Case presentation

A 17-year-old boy presented to our clinic for management of poor vision after congenital cataract surgery in the left eye. He had been diagnosed with congenital cataract in the left eye at 3 months of age (his medical history revealed an anterior subcapsular cataract), and he had undergone cataract extraction and IOL implantation in the left eye under general anesthesia at 6 months of age (the surgical method was recorded as cataract extraction combined with IOL implantation). Follow-up was performed once a month after the surgery. At 3 months postoperatively, the left eye exhibited a pinhole pupil, but the treating ophthalmologist chose to observe it without treatment. The patient thereafter received regular monocular occlusion treatment, starting with 6 h of prescribed daily patching and decreasing to 2 h of prescribed daily patching by the age of 6 years. During this period, the child’s guardian monitored the vision of the affected eye at home every week to ensure that the vision was not less than 0.5logMAR(20/40).Because the corrected vision couldn’t be improved, the affected eye had never had refractive corrections such as glasses or contact lenses.Subsequently, he began school and did not engage in any amblyopia training. There was no history of ocular or systemic genetic disease in the patient’s parents or immediate family members.

**Fig. 1:**
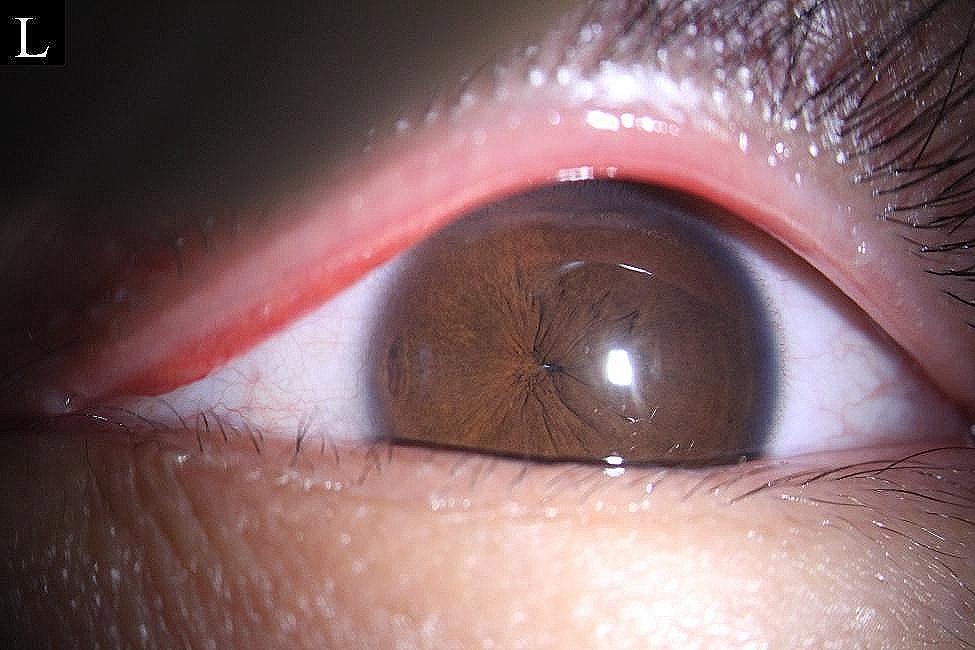
Preoperative slit-lamp examination for the left eye showed a pinhole pupil (∼ 0.5 mm)

Upon presentation to our clinic, the best corrected visual acuity (BCVA) in the patient’s right eye was 0.0 logMAR(20/20) with a refraction of − 5.75 diopters (D) cylinder/−2.25 D sphere, and the BCVA in his left eye was 0.5 logMAR(20/40) with a refraction of 0.0 D. Visual acuity in the left eye did not improve with correction; the intraocular pressure was 12 mmHg in the right eye and 15 mmHg in the left eye. Slit-lamp examination showed that the left cornea was transparent and the anterior chamber was deep. A pinhole pupil (approximately 0.5 mm) was evident in the left eye (Fig. 1), and its light reflex sensitivity was high (Video 1). Contractions of the iris sphincter and dilator muscle were clearly observed under light irradiation, but there were no responses to pupil-dilating drugs. The IOL was not visible. The number of endothelial cells was 2889/mm^2^ in the right eye and 2876/mm^2^ in the left eye. IOLMaster measurements showed that the axial length of the right eye was 27.02 mm. IOLMaster could not measure the left eye; A-ultrasound showed that the ocular axis was 24.21 mm. B-ultrasound revealed no obvious posterior segment abnormalities in the left eye. There were no obvious abnormalities in the anterior segment or fundus of the right eye.

**Fig. 2:**
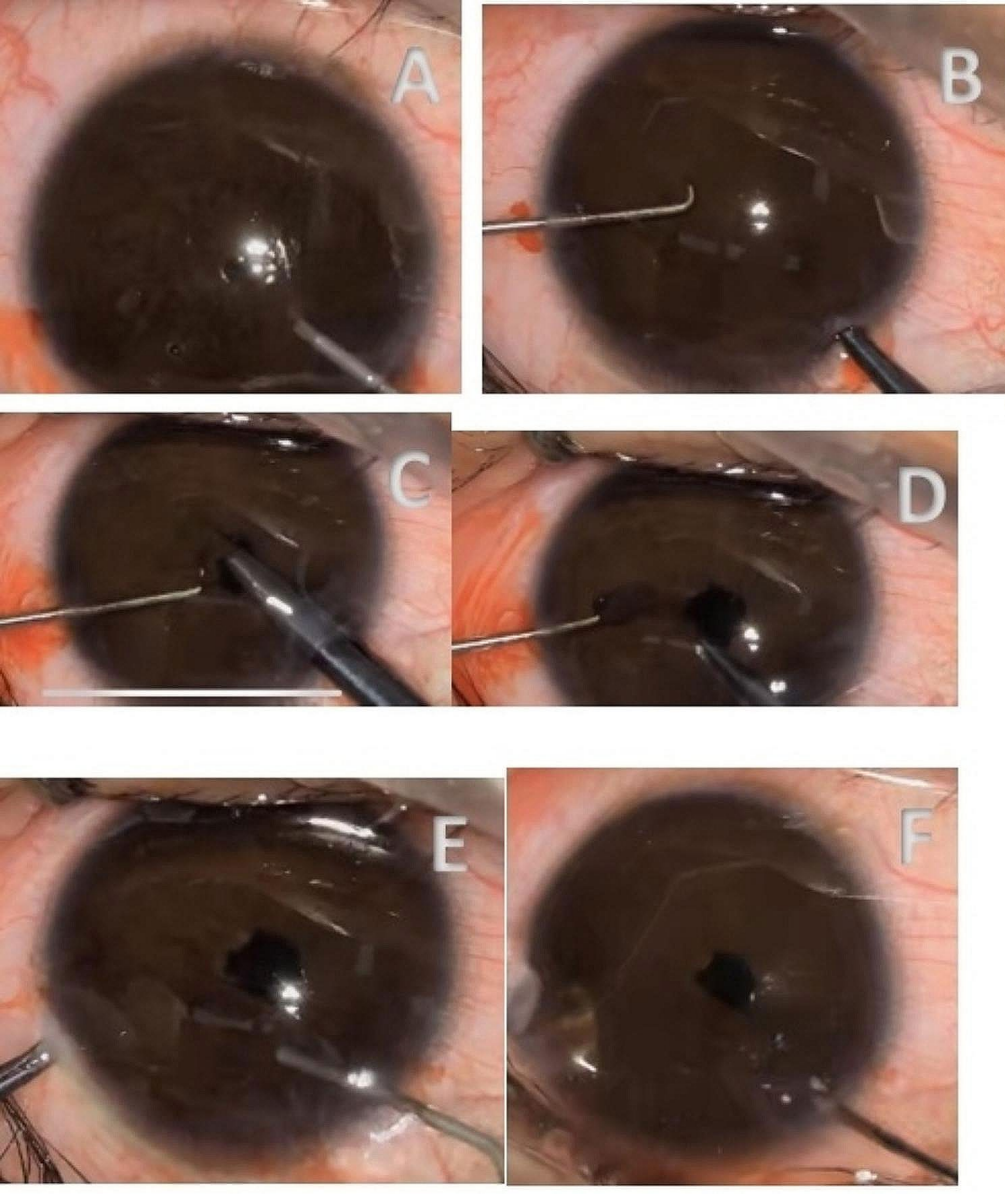
Clinical image of pupiloplasty. (**A**) viscoelastic had been injected into the anterior chamber; (**B**) Gently pull the iris with a retractor to expose the pupil; (**C**) the pinhole pupil was cut open with iris scissors; (**D**) a portion of the iris tissue was removed in a toroidal fashion to create a pupil approximately 3 mm in size; (**E**) The anterior chamber was flushed with balanced saline solution to remove the viscoelastic agent; (**F**) the corneal incision was closed by a water-tight approach

On June 30, 2023, left pupilloplasty was performed with the patient under corneal surface anesthesia. Considering the unpredictable status of the patient’s IOL, we prepared a 21 D IOL based on the A-ultrasound results in case of intraoperative IOL replacement. The surgical procedure proceeded in a manner that was considerably better than expected. After a clear corneal incision had been made and viscoelastic had been injected into the anterior chamber, the pinhole pupil was cut open with iris scissors; a portion of the iris tissue was removed in a toroidal fashion to create a pupil approximately 3 mm in size. The position of the IOL in the posterior part of the eye was confirmed to be correct, without dislocation or deviation. The anterior chamber was flushed with balanced saline solution to remove the viscoelastic agent, and the corneal incision was closed by a water-tight approach (Fig. 2).

**Fig. 3:**
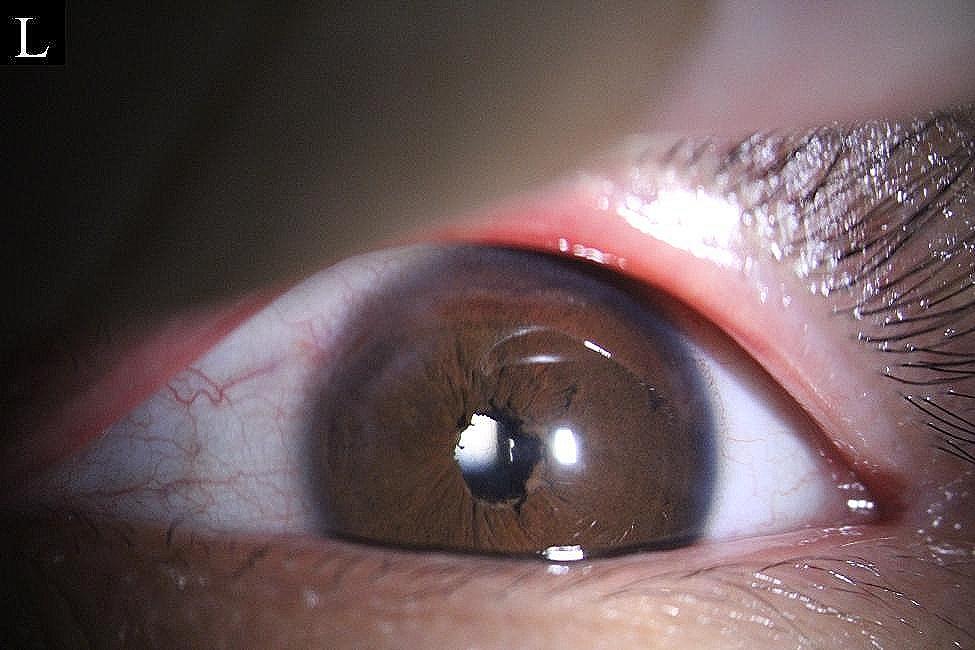
Postoperative slit-lamp examination for the left eye showed a central position with a diameter of ∼ 3 mm

**Fig. 4:**
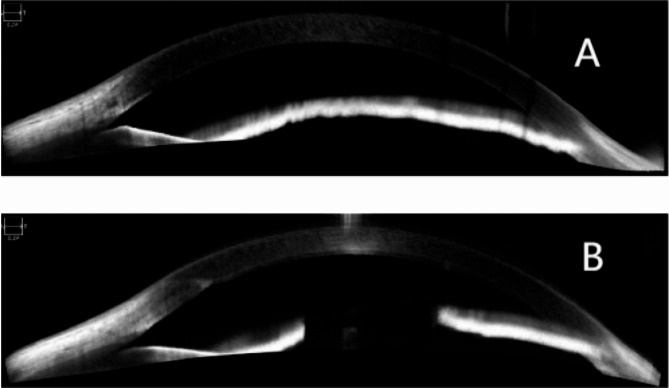
Preoperative and Postoperative as-oct of the left eye. (**A**) Preoperative as-oct couldn’t show a clear pupil. (**B**) Postoperative as-oct showed a central position with a diameter of ∼ 3 mm

**Fig. 5:**
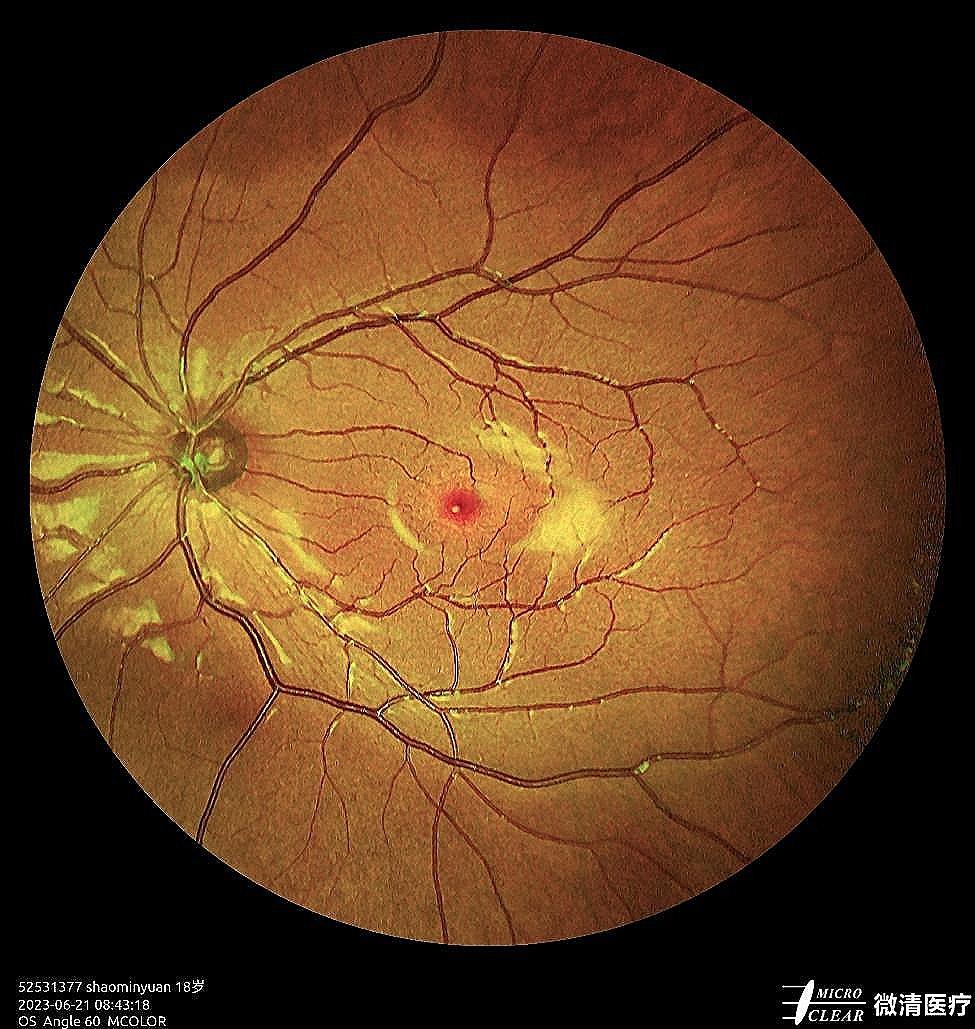
Postoperative fundas examination for the left eye revealed a reddish optic disc, good retinal vascular alignment, and a flat retina

**Fig. 6:**
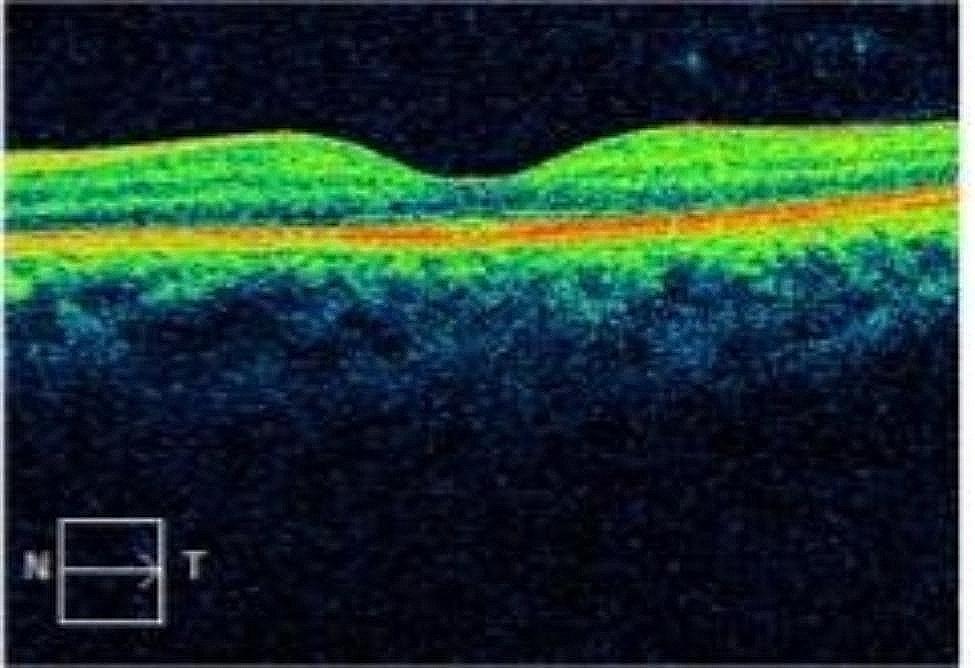
Optical coherence tomography showed that the macular structure in the left eye was normal

**Fig. 7:**
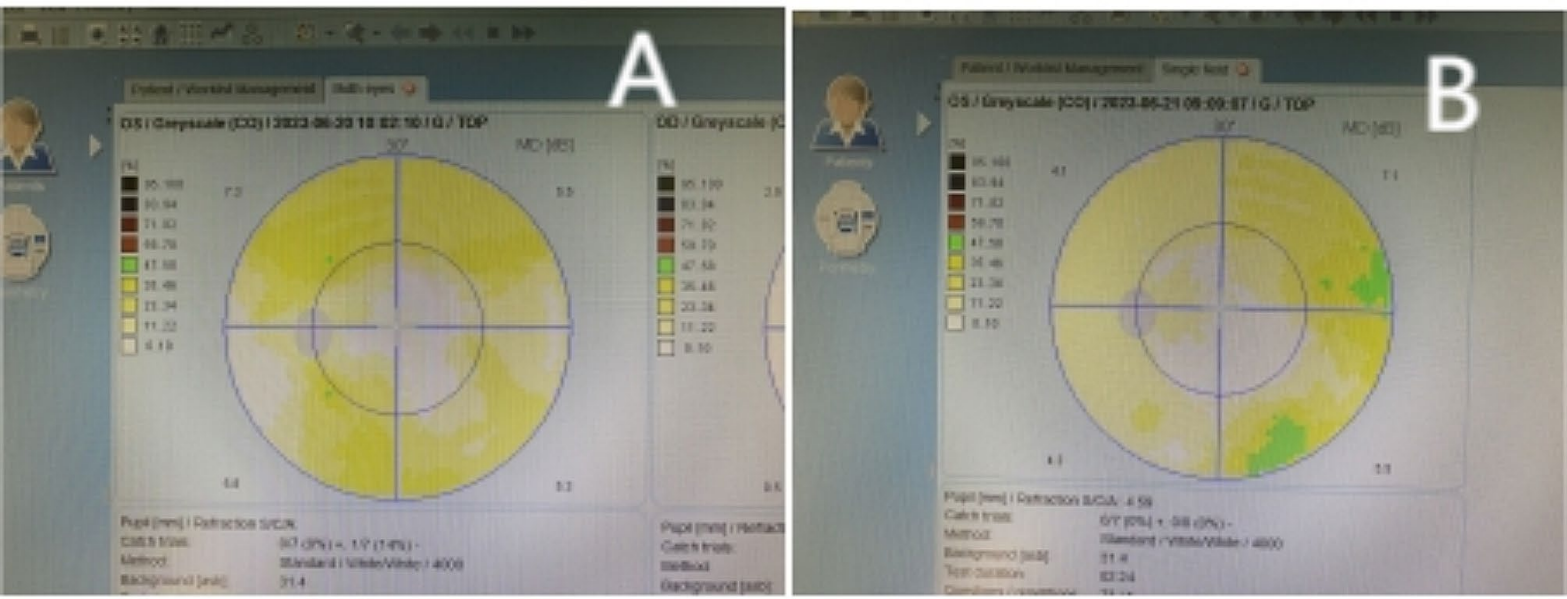
Preoperative (**A**) and Postoperative (**B**) visual field of the left eye demonstrated mild improvement

At the first follow-up on postoperative day 1, the uncorrected visual acuity in the patient’s left eye was 0.9 logMAR(20/100). The refraction results were − 2.23 D cylinder/−3.00 D sphere in the left eye, which achieved a BCVA of 0.0 logMAR(20/20). The intraocular pressure was 13 mmHg. The pupil was located in a central position with a diameter of approximately 3 mm (Figs. 3 and 4), and it showed light reflex sensitivity (Video 2). The IOL position was appropriate, and fundoscopy of the left eye was performed. This examination revealed a reddish optic disc, good retinal vascular alignment, and a flat retina (Fig. 5). Optical coherence tomography showed that the macular structure in the left eye was normal(Fig. 6). Additionally, comparison of the visual field in the patient’s left eye before and after surgery demonstrated considerable improvement (Fig. 7).

## Discussion and conclusions

Postoperative cataract complications in children mainly include PCO, aphakic or pseudophakic glaucoma, uveitis, pupillary displacement, and IOL decentration. Among these, PCO is the most common complication with an incidence of 60% [1, 4]. The most common pupillary-related complication after cataract surgery in children is pupil displacement [5]. Fibrinous membrane formation in the pupillary area is expected in children who undergo IOL implantation after congenital cataract extraction, and the associated inflammation can lead to posterior synechiae [4]. However, no reports to date have described the formation of a small pupil after congenital cataract surgery. We encountered a case of micropupil development without clinically significant posterior synechiae that occurred after the patient had undergone cataract phacoemulsification with IOL implantation during infancy. Some studies suggest that phacoemulsification-related intraoperative trauma to the iris and IOL induction are the main causes of pupillary abnormalities in patients with cataract [6]. In our patient, both damage to the iris secondary to intraoperative phacoemulsification and IOL-induced irritation after surgery may have contributed to micropupil formation.

Our team previously reported a case of congenital microcoria with extremely small pupil size (< 0.5 mm), severe amblyopia, and wasting exotropia. BCVA in the affected eye was 2.0 logMAR. The patient also underwent pupilloplasty, but the surgery did not lead to clinically significant improvement in visual acuity [7]. Microcoria, also known as congenital miosis, is an iris malformation that affects both regulation of the amount of light reaching the retina and the intraocular pressure. It is characterized by pinhole pupils (< 2 mm), iris hypopigmentation, and transillumination, causing both hemeralopia and light hypersensitivity and potentially leading to visual dysfunction or blindness [8]. Defective development of the iris musculature is considered to be the cause of this disease. Iris integrity is required to regulate the amount of light reaching the retina. Significant iris thinning with atrophy of the stroma and partial or total dilation inability have been observed among affected individuals [9]. Our patient with secondary microcoria also exhibited a pinhole pupil in the affected eye; unlike the patient with congenital microcoria, however, our patient had normal iris tissue, as well as normal pupillary muscles and high sensitivity to light reflexes. These factors may have allowed our patient’s retina to receive a variety of light stimuli, and they may help to explain the difference between the good visual prognosis of our patient and the poor visual prognosis of the previously described patient with microcoria.

The expectation of IOL implantation as opposed to contact lens correction centers on the provision of continuous and easily managed full-time optical correction along with amblyopia prevention [10]. Even so, primary IOL implantation in children aged < 6 months is controversial, and most surgeons recommend leaving children aphakic with further lens/spectacle correction [11]. Myopic shift (MS), resulting from both the natural physiological growth of the eye and any deviations that occur, is an important factor influencing the outcome of congenital cataract surgery [12]. Many studies have investigated MS in pseudophakic eyes and demonstrated that earlier IOL implantation is associated with greater MS [13]. Park et al. found significantly more severe postoperative MS in pseudophakic eyes than in the fellow phakic eyes after unilateral cataract surgery in patients aged < 6 years (− 3.25 ± 3.21 D vs. −1.78 ± 2.10 D) [14]. The Infant Aphakia Treatment Study revealed that eyes treated for monocular cataract in infancy, either with contact lenses or IOLs, exhibited axial growth similar to that of the fellow eyes despite having a shorter axial length at the time of surgery in 2017 [15]. In our patient, however, the affected eye not only retained good vision (BCVA of 0.5 logMAR(20/40) before pupilloplasty and 0.0 logMAR(20/20) after) but also had a significantly smaller degree of myopia and shorter axial length than the fellow eye (− 2.23 D cylinder/−3.00 D sphere vs. −5.75 D cylinder/−2.25 D sphere and 24.21 vs. 27.02 mm, respectively). These results differ from the typical outcome of monocular cataract surgery, where myopic drift causes the affected eye to develop a longer ocular axis. A small pupil diameter is well known to reduce stray incident light, which can help to improve visual acuity while reducing haze and halos. A small pinhole acts as a lens that focuses light, blocking light from the peripheral cornea and allowing the passage of central and paracentral rays. These dynamics enhance visual acuity and image quality by reducing aberrations throughout the optical system [16]. pinhole pupilloplasty was reportedly performed in patients with high astigmatism and higher-order aberrations. Their pupils were reduced to a mean diameter of approximately 1.43 ± 0.24 mm, and their postoperative uncorrected visual acuity was significantly improved (*P* < 0.001) [17]. Is it possible that the micropupil in this case actually improved the visual quality of the affected eye, leading to the better ocular development than in the fellow eye? Another consideration is that the patient received perennial monocular occlusion treatment after cataract surgery at 3 months of age, and the early visual deprivation of the other eye may have induced axial elongation and worsening of myopia. Overuse of the other eye may have also increased the degree of myopia.

We have described a case of micropupil formation secondary to congenital cataract surgery favoring the development of the affected eye. Such a complication involving micropupil formation after congenital cataract surgery has not been reported in the past, and this case increases our understanding of the complications that may occur after congenital cataract surgery. The patient in this case had a small pupil but healthy iris tissue and a sensitive pupillary light reflex, which differs from congenital microcoria and may have been the basis for his good visual development. This highlights the important role of the pupillary light reflex in visual development. In this rare case, we also incidentally observed a beneficial effect of a small pupil size on visual development. This phenomenon is difficult to study in routine clinical practice, and we believe that it may offer valuable insights for further clinical research.

Our case has some limitations, such as its rarity and uniqueness; additionally, some details could not be analyzed because of the long duration of the patient’s diagnosis and treatment. In future studies involving children who have undergone congenital cataract surgery, we will focus on observing and documenting pupillary-related complications and investigate whether interventions targeting pupil changes or incorporating small holes can reduce the visual interference of the affected eye and increase the possibility of good visual development.

### Electronic supplementary material

Below is the link to the electronic supplementary material.

Supplementary Material 1

Supplementary Material 2

## Data Availability

No datasets were generated or analysed during the current study.

## Abbreviations

BCVA: Best-corrected visual acuity
PCO: Posterior capsule opacification
IOL: Intraocular lens
D: Diopter
MS: Myopic shift

## References

[1] Chen J, Chen Y, Zhong Y, Li J (2020). Comparison of visual acuity and complications between primary IOL implantation and aphakia in patients with congenital cataract younger than 2 years: a meta-analysis. J Cataract Refract Surg.

[2] Boothe RG, Dobson V, Teller DY (1985). Postnatal development of vision in human and nonhuman primates. Annu Rev Neurosci.

[3] Angée C, Nedelec B, Erjavec E, Rozet JM, Fares Taie L. Congenital Microcoria: clinical features and Molecular Genetics. Genes (Basel). 2021;12(5). 10.3390/genes12050624 PMC8143514 33922078

[4] Chan WH, Biswas S, Ashworth JL, Lloyd IC (2012). Congenital and infantile cataract: aetiology and management. Eur J Pediatr.

[5] Bremond-Gignac D, Daruich A, Robert MP, Valleix S (2020). Recent developments in the management of congenital cataract. Ann Transl Med.

[6] Yap EY, Aung T, Fan RF. Pupil abnormalities on the first postoperative day after cataract surgery. Int Ophthalmol. 1996-1997;20(4):187–92. 10.1007/BF00175258 9112185

[7] Hao L, Ma Z, Song C, Zhu S (2022). A rare case of congenital pupillary abnormality: a case report. BMC Ophthalmol.

[8] Pozza E, Verdin H, Deconinck H, Dheedene A, Menten B, De Baere E, Balikova I (2020). Microcoria due to First Duplication of 13q32.1 including the GPR180 gene and maternal mosaicism. Eur J Med Genet.

[9] Jeeva-Patel T, Lutchman C, Margolin E (2021). Congenital Microcoria. Ophthalmology.

[10] Crouch ER, Crouch ER, Pressman SH (2002). Prospective analysis of pediatric pseudophakia: myopic shift and post operative outcomes. J AAPOS.

[11] Weakley DR, Lynn MJ, Dubois L, Cotsonis G, Wilson ME, Buckley EG (2017). Myopic shift 5 years after intraocular lens implantation in the infant aphakia treatment study. Ophthalmology.

[12] Liu Z, Long E, Chen J (2016). Developmental profile of ocular refraction in patients with congenital cataract: a prospective cohort study. Lancet.

[13] Weakley DR, Lynn MJ, DuBois LG (2017). Myopic shift 5 years after intraocular lens implantation in the infant aphakia treatment study. Ophthalmology.

[14] Park Y, Yum HR, Shin SY, Park SH (2022). Ocular biometric changes following unilateral cataract surgery in children. PLoS ONE.

[15] Wilson ME, Trivedi RH, Weakley DR, Cotsonis GA, Lambert SR, Infant Aphakia Treatment Study Group (2017). Globe Axial length growth at Age 5 years in the infant Aphakia Treatment Study. Ophthalmology.

[16] Narang P, Agarwal A, Ashok Kumar D, Agarwal A (2019). Pinhole pupilloplasty: small-aperture optics for higher-order corneal aberrations. J Cataract Refract Surg.

[17] Narang P, Holladay J, Agarwal A, Jaganathasamy N, Kumar DA, Sivagnanam S (2019). Application of Purkinje images for pinhole pupilloplasty and relevance to chord length mu. J Cataract Refract Surg.

